# 450. Type I Interferon Autoantibodies Are Detected in Those with Critical COVID-19, Including a Young Female Patient

**DOI:** 10.1093/ofid/ofab466.649

**Published:** 2021-12-04

**Authors:** Debra Yee, Marana Tso, Elana Shaw, Lindsey B Rosen, Emily Samuels, Paul Bastard, Jean-Laurent Casanova, Steven M Holland, Helen C Su, Stephanie A Richard, Katrin Mende, Tahaniyat Lalani, David A Lindholm, David A Lindholm, Mark P Simons, David Tribble, Allison Malloy, Eric Laing, Brian Agan, Simon Pollett, Timothy Burgess, Andrew L Snow

**Affiliations:** 1 Uniformed Services University of the Health Sciences, Bethesda, Maryland; 2 National Institute of Allergy and Infectious Diseases (NIAID), National Institutes of Health, Bethesda, Maryland; 3 The Rockefeller University, New York, New York; 4 Laboratory of Clinical Immunology and Microbiology, National Institute of Allergy and Infectious Diseases, National Institutes of Health, Bethesda, Maryland; 5 Immune Deficiency Genetics Section, Laboratory of Clinical Immunology and Microbiology, National Institute of Allergy and Infectious Diseases, National Institutes of Health, Bethesda, Maryland; 6 IDCRP, Rockville, Maryland; 7 Infectious Disease Clinical Research Program, Department of Preventive Medicine and Biostatistics, Uniformed Services University of the Health Sciences and Brooke Army Medical Center, JBSA Fort Sam Houston, TX, San Antonio, TX; 8 Infectious Disease Clinical Research Program, Bethesda, MD, The Henry M. Jackson Foundation, Bethesda, MD, and Naval Medical Center Portsmouth, VA, Portsmouth, Virginia; 9 Uniformed Services University of the Health Sciences; Brooke Army Medical Center, San Antonio, TX; 10 Walter Reed National Military Medical Center, Bethesda, Maryland; 11 Infectious Disease Clinical Research Program, USU/HJF, Bethesda, Maryland; 12 Infectious Disease Clinical Research Program, Bethesda, Maryland

## Abstract

**Background:**

Approximately 10-20% of patients with critical COVID-19 harbor neutralizing autoantibodies (auto-Abs) that target type I interferons (IFN), a family of cytokines that induce critical innate immune defense mechanisms upon viral infection. Studies to date indicate that these auto-Abs are mostly detected in men over age 65.

**Methods:**

We screened for type I IFN serum auto-Abs in sera collected < 21 days post-symptom onset in a subset of 103 COVID-19 inpatients and 24 outpatients drawn from a large prospective cohort study of SARS-CoV-2 infected patients enrolled across U.S. Military Treatment Facilities. The mean age of this n = 127 subset of study participants was 55.2 years (SD = 15.2 years, range 7.7 – 86.2 years), and 86/127 (67.7%) were male.

**Results:**

Among those hospitalized 49/103 (47.6%) had severe COVID-19 (required at least high flow oxygen), and nine subjects died. We detected neutralizing auto-Abs against IFN-α, IFN-ω, or both, in four inpatients (3.9%, 8.2% of severe cases), with no auto-Abs detected in outpatients. Three of these patients were white males over the age of 62, all with multiple comorbidities; two of whom died and the third requiring high flow oxygen therapy. The fourth patient was a 36-year-old Hispanic female with a history of obesity who required mechanical ventilation during her admission for COVID-19.

**Conclusion:**

These findings support the association between type I IFN auto-antibody production and life-threatening COVID-19. With further validation, reliable high-throughput screening for type I IFN auto-Abs may inform diagnosis, pathogenesis and treatment strategies for COVID-19, particularly in older males. Our finding of type I IFN auto-Ab production in a younger female prompts further study of this autoimmune phenotype in a broader population.

**Disclosures:**

**David A. Lindholm, MD**, American Board of Internal Medicine (Individual(s) Involved: Self): Member of Auxiliary R&D Infectious Disease Item-Writer Task Force. No financial support received. No exam questions will be disclosed ., Other Financial or Material Support **David Tribble, M.D., DrPH**, **Astra Zeneca** (Other Financial or Material Support, HJF, in support of USU IDCRP, funded under a CRADA to augment the conduct of an unrelated Phase III COVID-19 vaccine trial sponsored by AstraZeneca as part of USG response (unrelated work)) **Simon Pollett, MBBS**, **Astra Zeneca** (Other Financial or Material Support, HJF, in support of USU IDCRP, funded under a CRADA to augment the conduct of an unrelated Phase III COVID-19 vaccine trial sponsored by AstraZeneca as part of USG response (unrelated work))

